# Effect of 3-nitrooxypropanol on enteric methane emissions of feedlot cattle fed with a tempered barley-based diet with canola oil

**DOI:** 10.1093/jas/skad237

**Published:** 2023-07-10

**Authors:** Amelia K Almeida, Frances Cowley, Joe P McMeniman, Alex Karagiannis, Nicola Walker, Luis F M Tamassia, Joseph J McGrath, Roger S Hegarty

**Affiliations:** School of Environmental and Rural Science, University of New England, Armidale, NSW 2351, Australia; School of Agriculture and Environment, Massey University, Palmerston North 4442, New Zealand; School of Environmental and Rural Science, University of New England, Armidale, NSW 2351, Australia; Feedlot Program, Meat and Livestock Australia Limited (MLA), North Sydney, NSW 2060, Australia; Animal Nutrition and Health, DSM Nutritional Products, Wurmisweg 576 4303, Kaiseraugst, Switzerland; Animal Nutrition and Health, DSM Nutritional Products, Wurmisweg 576 4303, Kaiseraugst, Switzerland; Animal Nutrition and Health, DSM Nutritional Products, Wurmisweg 576 4303, Kaiseraugst, Switzerland; School of Environmental and Rural Science, University of New England, Armidale, NSW 2351, Australia; Animal Nutrition and Health, DSM Nutritional Products, Wurmisweg 576 4303, Kaiseraugst, Switzerland; School of Environmental and Rural Science, University of New England, Armidale, NSW 2351, Australia

**Keywords:** carbon neutral, feedlot, methane, rumen fermentation, sustainability, 3-NOP

## Abstract

A dose-response experiment was designed to examine the effect of 3-nitrooxypropanol (3-NOP) on methane (CH_4_) emissions, rumen function and performance of feedlot cattle fed a tempered barley-based diet with canola oil. Twenty Angus steers of initial body weight (BW) of 356 ± 14.4 kg were allocated in a randomized complete block design. Initial BW was used as the blocking criterion. Cattle were housed in individual indoor pens for 112 d, including the first 21 d of adaptation followed by a 90-d finishing period when five different 3-NOP inclusion rates were compared: 0 mg/kg dry matter (DM; control), 50 mg/kg DM, 75 mg/kg DM, 100 mg/kg DM, and 125 mg/kg DM. Daily CH_4_ production was measured on day 7 (last day of starter diet), day 14 (last day of the first intermediate diet), and day 21 (last day of the second intermediate diet) of the adaptation period and on days 28, 49, 70, 91, and 112 of the finisher period using open circuit respiration chambers. Rumen digesta samples were collected from each steer on the day prior to chamber measurement postfeeding, and prefeeding on the day after the chamber measurement, for determination of rumen volatile fatty acids (VFA), ammonium-N, protozoa enumeration, pH, and reduction potential. Dry matter intake (DMI) was recorded daily and BW weekly. Data were analyzed in a mixed model including period, 3-NOP dose and their interaction as fixed effects, and block as a random effect. Our results demonstrated both a linear and quadratic (decreasing rate of change) effect on CH_4_ production (g/d) and CH_4_ yield (g/kg DMI) as 3-NOP dose increased (*P* < 0.01). The achieved mitigation for CH_4_ yield in our study ranged from approximately 65.5% up to 87.6% relative to control steers fed a finishing feedlot diet. Our results revealed that 3-NOP dose did not alter rumen fermentation parameters such as ammonium-N, VFA concentration nor VFA molar proportions. Although this experimental design was not focused on the effect of 3-NOP dose on feedlot performance, no negative effects of any 3-NOP dose were detected on animal production parameters. Ultimately, the knowledge on the CH_4_ suppression pattern of 3-NOP may facilitate sustainable pathways for the feedlot industry to lower its carbon footprint.

## Teaser

Rumen fermentation, methane (CH_4_), and production parameters were investigated in feedlot cattle supplemented with increasing inclusion rates of 3-nitrooxypropanol. Our results revealed a potential CH_4_ abatement of up to 90% during a 90-d finishing period on a tempered barley diet containing monensin and 7% lipid content.

## Introduction

Reducing greenhouse gas (GHG) production is an imperative set by the Paris Agreement, in which nations committed to maintaining increases in global average temperature of less than 2 °C above the pre-industrialization levels (UNFCCC, 2015). In this scenario, mitigating enteric methane (CH_4_) from ruminant livestock production presents as a great opportunity for meeting these climate stabilization targets, since as the main non-CO_2_ GHG, CH_4_ has a relatively brief lifetime in the atmosphere (~9 yr; Ripple et al., 2014). As the main GHG from agriculture, livestock enteric CH_4_ contributes to 11.6% of global GHG emissions from anthropogenic activities (Ripple et al., 2014; Herrero et al., 2016).

As a method to mitigate enteric CH_4_ production, 3-nitrooxypropanol (3-NOP) is a promising nutritional strategy. This compound is a structural analogue of the nickel enzyme methyl CoM reductase produced by the methanogenic archaea, and inhibits the last step of CH_4_ formation in the rumen (Duin et al., 2016). In the rumen, 3-NOP is highly soluble and is first oxidized to 3-nitrooxypropionic acid, which is then hydrolyzed to 3-hydroxypropionic acid and low concentrations of inorganic nitrate (Thiel et al., 2019). The 3-hydroxypropionic acid may then be used by mammalian cells for synthesis of gluconeogenesis substrates such as propanoyl-CoA. As 3-NOP is metabolized rapidly, when supplemented within the recommended dose range, it does not accumulate in animal’s tissues or blood stream (Thiel et al., 2019) and therefore seemingly poses little risk to consumers of animal products.

Among ruminants, dietary supplementation of 3-NOP yielded a mean reduction in CH_4_ yield of 30%, across a variety of ruminant diets (Almeida et al., 2021). To this date, studies have shown that 3-NOP delivers a potential CH_4_ yield suppression of up to 76 to 81% for feedlot finishing diets (Vyas et al., 2016; Alemu et al., 2021a). Previous studies indicate that feeding 3-NOP to feedlot cattle does not affect feed digestibility (Romero-Perez et al., 2014; Zhang et al., 2021), and may improve feed efficiency (Vyas et al. 2016, Vyas et al., 2018a; Alemu et al., 2021b), with a decrease in DM intake in feedlot cattle (Vyas et al. 2016, Vyas et al., 2018a; Alemu et al. 2021a, 2021b; Zhang et al., 2021).

Based on the existing literature, we hypothesized that a dose-response pattern of CH_4_ reduction will be observed when feeding feedlot cattle with increasing inclusion rates of 3-NOP, which may be accompanied by a reduction in feed intake and a shift in ruminal fermentation. Therefore, the objective of this study was to evaluate the opportunity to suppress CH_4_ in feedlot cattle through inclusion of increasing inclusion rates of 3-NOP in the diet. Knowledge regarding the CH_4_ suppression pattern of 3-NOP may facilitate sustainable pathways for the feedlot industry to lower its carbon footprint.

## Materials and Methods

The Animal Ethics Committee of the University of New England approved all procedures involved in this experiment (Authority no.: AEC-20-061).

### Animals, diets, and experimental design

Twenty Angus steers with initial body weight (BW) of 356 ± 14.4 kg and age 15 to 18 mo were sourced from a single property and transported to the Large Animal Facility at the Centre for Animal Research and Teaching (CART) of the University of New England (Armidale, NSW Australia). Prior to the experiment, all animals were fitted with a visual eartag, vaccinated for clostridial organisms and leptospirosis (Ultravac 7‐in‐1, Zoetis, Melbourne, Australia); Mannheimia haemolytica and Bovine herpes virus (Bovilis MH + IBR, Coopers Animal Health, Macquarie Park, NSW, Australia) and treated with a pour-on anthelmintic (Cydectin plus Fluke: Virbac, Milperra NSW, Australia). Steers had no hormonal growth implants administered. A week prior to commencement of the experiment, steers were allocated into two blocks of 10 steers, according to BW (light and heavy), and were allowed 7 d to acclimate to their pens and housing on a roughage diet (100% oaten hay to ad libitum). Each steer was housed in an individual indoor pen in the CART facility (3 × 2 m, cement flooring and provided with rubber mats). Within each block, the steers were randomly allocated to treatments consisting one of five inclusion rates of dietary 3-NOP in the form of “Bovaer 10” (a blend of 3-NOP included at a minimum of 10% on a carrier of silicate and dried propylene glycol, DSM Nutritional Products AG, Basel, Switzerland): 0 mg/kg dry matter (DM; control), 50, 75, 100, or 125 mg/kg DM in the finisher period. Treatment premixes were balanced to the same DM inclusion rate as the 125 mg 3-NOP/kg DM treatment by addition of extra carrier (silicate and dried propylene glycol, Table 1). The control included a placebo of silicate and dried propylene glycol only. The second cohort commenced the experimental period one day after the first cohort to allow staggered measurement of CH_4_ emissions in respiration chambers (*N* = 10).

**Table 1.** Composition of feedlot total mixed rations feed to Angus steers during the 112-d feeding period

Item	Diet (feeding days)^1^			
	Starter (0 to 7)	Intermediate I (8 to 14)	Intermediate II (15 to 21)	Finisher (22 to 112)
Ingredient, % DM				
Tempered Barley^2^	35.27	49.89	64.48	80.75
Cereal hay	23.97	16.93	9.85	–
Oaten chaff	12.16	8.59	5.00	3.11
Whole cottonseed	12.17	11.25	10.31	9.35
Mill run	10.45	7.09	3.64	–
Molasses	3.44	2.88	2.38	1.79
Canola oil	–	0.81	1.75	2.39
Treatment mix^3^	0.04	0.06	0.09	0.11
Dry supplement ^4^	2.50	2.50	2.50	2.50
Chemical composition (DM-basis)^5^				
Moisture, % DM	12.5	12.6	13.3	19.9
Organic matter, % DM	94.0	94.0	95.25	96.2
Ash, % DM	6.00	6.00	4.75	3.80
Crude protein, % DM	13.9	13.8	14.4	15.1
Fat, % DM	4.35	4.70	5.58	6.66
Carbohydrates, % DM	12.2	10.9	7.73	4.44
NDF, % DM	40.5	33.7	31.3	29.0
Starch, % DM	20.0	28.3	35.7	42.3
GE, MJ/kg DM	18.6	18.7	18.8	19.3
ME, MJ/kg DM	11.5	12.2	12.9	13.8

^1^Diets were formulated using the Concept 5 software, targeting ADG of 2 kg according to NASEM (2016).

^2^Barley was rolled to a target density of 45 kg/hL and 20% of moisture after being steeped for 20 h, no surfactant was added.

^3^3-NOP was added to the experimental diets in a way that the placebo (propylene glycol adsorbed on silicic acid, precipitated and dried) + Bovaer 10 (minimum 10% 3-NOP) added up to the same DM inclusion in the diet.

^4^Supplied per kilogram of supplement (DM basis) 6.0 mg cobalt (MICROGRAN Co 5% BMP), 400 mg copper (Copper Sulphate Pentahydrate, 20mg iodine (Calcium iodate anhydrous 63%), 800 mg manganese (Manganese Sulphate 31%), 4 mg selenium (MICROGRAN Se 4.5% BMP), 2.4 g zinc (Zinc Sulphate Monohydrate 35%), 2.0% magnesium (Magnesium Oxide 54%), 22.0% calcium (calcium carbonate), 2.0% sulfur (Calcium Sulphate), 10% salt (feed grade salt), 11% urea, 1 g monensin (Rumensin 200), 88 KIU vitamin A (ROVIMIX A 1000), 11 KIU vitamin D (ROVIMIX D3-500) and 1.1 KIU vitamin E (ROVIMIX E-50 Adsorbate).

^5^analyzed for content of crude protein (AOAC Method 2001.11), ADF (AAFCO Method 008.08), NDF (NFTA Method 2.2.2.5), organic matter (ISO 5984:2002(E)), ether extract (AFIA Method 1.14R), starch (AOAC Method 996.11), water soluble carbohydrates (AFIA Method 1.11A), (NSW DPI Laboratory Services—Wagga Wagga Chemistry Services Laboratory, Wagga Wagga). ME content was calculated according to the equations for grains and concentrates of AFIA Method 2.2R (MAFF, 1990); all diets contained 25 ppm of monensin.

**Figure 1.**
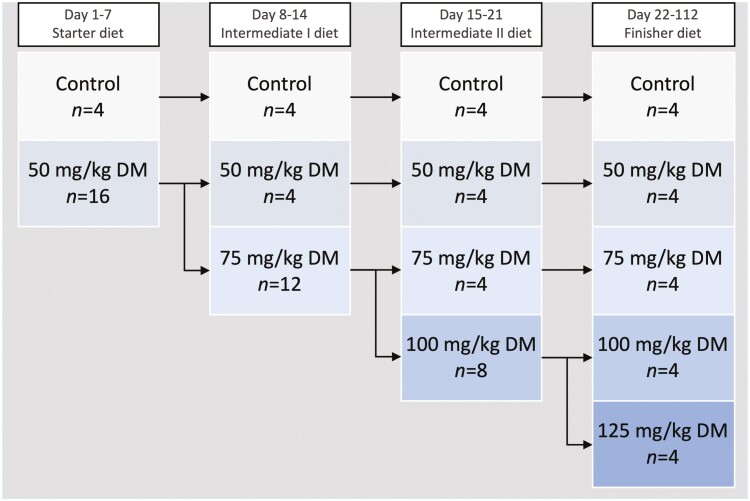
Trial design depicting the transition to the high-grain diet and 3-NOP dose (CH_4_ measurements on days 7, 14, and 21) and finisher period (CH_4_ measurements on days 28, 49, 70, 91 and 112).

The experimental feeding period for both groups was 112 d, which was split into an adaptation period (days 0 to 21) and a finisher period (days 22 to 112; Figure 1). A 21-d period of adaptation to a high grain-content diet, through a starter and two intermediate grain-content diets preceded the finisher period, to permit adjustment of animal eating behavior and rumen microflora in order to avoid risks of acidosis (Table 1). This reflected commercial feedlot practice, with all diets containing 25 mg/kg DM of monensin, as standard for Australian management systems. During the adaptation period, the diet was transitioned gradually from 35.3% to 80.8% tempered barley (DM basis; Table 1) in a step-wise manner. The finisher period diet was fixed at 80.8 % tempered barley, and was typical of an Australian feedlot finisher diet. Additionally, the adaptation protocol progressively increased the 3-NOP inclusion rate in the diets by increments of 25 mg/kg DM every 7 d to the final treatment inclusion rate (from days 0 to 21), to allow for rumen adaptation. The control steers (*N* = 4) received the placebo premix across each adaptation step (Table 1). The remaining steers (*N* = 16) were assigned to one of four 3-NOP inclusion rates (50, 75, 100, and 125 mg/kg DM) received 50 mg of 3-NOP/kg DM from days 0 to 7. From days 8 to 14, steers (*N* = 12) assigned to 3-NOP inclusion rates 75, 100, and 125 mg/kg DM were progressively increased to receive 75 mg of 3-NOP (Figure 1). From days 15 to 21, steers (*N* = 8) assigned to 3-NOP inclusion rates 100 and 125 mg/kg DM were progressively increased to receive 100 mg of 3-NOP (Figure 1). By the end of the adaptation period (day 22), four steers were receiving one of each of the five dietary treatments: control, 50, 75, 100, or 125 mg 3-NOP/kg DM (Figure 1). During the adaptation period, the diets were offered at increasing multiples of maintenance energy requirement (MER), estimated according to the NASEM (2016). After the adaptation period, all animals were fed with 2.7 × MER once daily at 0800 hours (block 1), and 0900 hours (block 2). All steers had unrestricted access to clean water at all times. Offered feed and refusals were recorded daily and sampled for DM by oven drying at 65 °C. Individual BW (nonfasted) was measured 1 h before feeding on days 0, 6, 13, 20, 27, 48, 69, 90, 111 and 3 h after feeding on days 8, 15, 22, 29, 50, 71, 92, 113 on a cattle scale with Wedderburn loadbars and a Gallagher read-out unit (Gallagher W310, Gallagher, Hamilton, New Zealand). Prior to each weigh day the scale was checked with 150 kg of weights verified with a second scale.

### Measurements of methane production

Daily CH_4_ production was estimated on day 7 (last day of starter diet), day 14 (last day of the first intermediate diet), and day 21 (last day of the second intermediate diet) of the adaptation period and on d days 28, 49, 70, 91, and 112 (five sampling periods) of the finisher period. For this purpose, the steers were confined over 23 h in open circuit respiration chambers. Chambers were sealed at 0800 hours (block 1) or 0900 hours (block 2), when steers were fed. Air temperature was controlled centrally for all chambers. Air temperature and relative humidity were measured in each chamber using sensors (BME280, Bosch Sensortec, Gerlingen, Germany). The average temperature and humidity throughout the experiment within chamber was 19.2 ± 3.38 and 78.7 ± 9.07, respectively. Air flow through each chamber (mean = 1.6 m^3^/min) was measured using a flow meter (Model ST75V, Fluid Components International, San Marcos, CA, USA). The concentrations of CH_4_, CO_2_, and O_2_ were measured in the chamber incoming (ambient) and exhaust air streams using a Servomex Multigas Analyser (Servomex 4100 Gas Purity Analyser) calibrated for these gases. Moisture was removed by a Nafion drying column before a multiplexer was used to direct the dried sample air from each chamber and the ambient air into the analyzer in turn. CH_4_, CO_2_, and O_2_ concentrations were measured over 10 s after a 40 s purge time, by the Servomex analyzer. Air flow and gas concentration data from the sampled air were loaded directly into a daily workbook with separate Excel spreadsheets for each chamber every 9 min, and used to calculate g of CH_4_/L air. CH_4_ production was averaged hourly and daily CH_4_ production estimated by the area under the curve by the approximate integral using trapezoidal rule. Recovery of CH_4_ through the chambers was assessed premeasurement and postmeasurement by introducing pure CH_4_ at a known rate via a mass flow controller (Smart Trak 2 Series 100, Sierra Instruments, Monterey, CA, USA) and the Servomex analyzer was used to quantify CH_4_ concentration. All daily CH_4_ emission data was corrected for 100% of CH_4_ recovery (mean 92.6% ± 1.36%). The recovery lower than 100% would not affect the detection of any treatment effects. Please refer to Hegarty et al. (2014) for further details on the use of the open-circuit respiration chambers and CH_4_ measurements protocols.

### Measurement of rumen function

Postfeeding rumen digesta samples were collected from each steer 4 h after feeding on day 6 (Starter), day 13 (Intermediate 1), day 20 (Intermediate 2) of the adaptation period, and on days 27, 48, 69, 90, and 111 of the finisher periods, by esophageal intubation. Likewise, prefeeding rumen digesta samples were collected from each steer 1 h prior to feeding on day 8 (Starter), day 15 (Intermediate 1), day 23 (Intermediate 2) of the adaptation period, and on day 27, 48, 69, 90, and 111 of the Finisher periods. Immediately after sampling, rumen fluid was measured for reduction potential (Mettler Toldeo SevenEasy S20 pH meter with TPS Intermediate Junction Redox Sensor, Port Melbourne, Victoria, Australia) and pH (EcoScan Portable pH/ORP meter with TPS pH Sensor, Eutech Instruments, Pte Ltd, Singapore) after proper calibration of both devices, and then subsampled for microscopic enumeration of ciliate protozoa (4 mL subsample preserved with 16 mL isotonic formaldehyde-saline) and analysis of fermentation metabolites (~15 mL subsampled and acidified with five drops of concentrated H_2_SO_4_ and frozen at −18 °C for analysis).

Volatile fatty acids (VFA) were determined by gas chromatography (Nolan et al., 2010). Rumen ammonia-N was determined by Skalar methodology, based on the modified Berthelot reaction (de Raphelis et al., 2016). For protozoa enumeration, a subsample of the diluted rumen fluid was stained with brilliant green (Nguyen and Hegarty, 2016) prior to microscopic enumeration of ciliate protozoa on a Fuchs—Rosenthal optical counting chamber (0.0625 mm^2^, 0.2 mm depth) using a technique adapted from Dehority (1984).

### Sampling and chemical analysis of feeds

From each treatment feed mix, a representative sample was collected daily. DM content was determined on a ~150 g subsample by oven drying at 65 °C, until there was no change in weight. A further ~150 g subsample was bulked weekly and analyzed for content of crude protein (AOAC, 1990; Method 2001.11), acid detergent fiber (ADF, AAFCO Method 008.08), neutral detergent fiber (NDF, NFTA Method 2.2.2.5), organic matter (ISO 5984:2002(E)), ether extract (AFIA Method 1.14R), starch (AOAC, 1990; Method 996.11), water soluble carbohydrates (AFIA Method 1.11A), digestible dry and organic matter (AFIA Method 1.7R; NSW DPI Laboratory Services—Wagga Wagga Chemistry Services Laboratory, Wagga Wagga, NSW—analyzed chemical composition shown in Supplementary Material Table S1). Metabolizable energy (ME) content was calculated according to the equations for grains and concentrates of AFIA Method 2.2R (MAFF, 1990). Additionally, during every mixing event a sample of feed was collected and bulked by diet and analyzed for 3-NOP (van Gastelen et al., 2020). Fatty acid profile of the oil used in this trial was also performed (ISO/IEC 17025 – 2017; Supplementary Material Table S2).

### Statistical analyses

The adaptation period was required for functional adaptation of the animal and their rumen microbiota to the experimental diet and 3-NOP doses. However, it generates a confounding aspect on evaluated variables: time and diets (including 3-NOP inclusion rate) and so the experimental design was not analyzed considering the 112-d period altogether. In this regard, descriptive analysis (PROC MEANS, SAS Inst., Cary, NC, USA) was performed and reported for the adaptation period (days 1 to 21), and the finisher period was analyzed separately (days 22 to 112).

A mixed model was implemented, including sampling period, 3-NOP inclusion rate and their interaction as a fixed effect, and block as a random effect. All variables (*Y*) during the finishing period were analyzed considering the following statistical model:


$${\text{Y}}_{\text{ijkl}}=\text{μ}+{\text{N}}_{\text{i}}+{\text{T}}_{\text{i}}+{\text{NT}}_{\text{ij}}+{\text{b}}_{\text{k}}+{\text{e}}_{\text{ijkl}}$$

where μ = the overall mean,

N_i_ = the effect of the ith 3-NOP inclusion rate (0, 50, 75, 100, or 125 mg/kg DM)

T_j_ = the effect of the jth sampling period (j = 1, …, 5),

NT_ij_ = the interaction between the ith 3-NOP inclusion rate and the jth period

b_k_ = the effect of the kth block, and

e_ijkl_ = the random error associated with the lth steer of the ith 3-NOP inclusion rate in the jth sampling period in block k, e ~ N(0, σ^2^_e_).

When 3-NOP inclusion rate was found significant, orthogonal contrasts were used to evaluate a response curve of 3-NOP inclusion rate. The error correlation matrix due to the repeated measures (sampling period) was modeled as unstructured, as it yielded the smallest corrected Akaike Information Criterion of a range of error structures tested. PROC IML of SAS was used to generate linear and quadratic co-efficients for the unequally spaced contrasts (0, 50, 75, 100, 125). Linear and quadratic regression lines were fitted in PROC MIXED using the CONTRAST statement. Statistical significance was declared at *P* < 0.05. Differences between means were determined using the P‐DIFF option of the LSMEANS statement, which is based on Fisher’s F‐protected least significant difference test.

## Results

### Methane emissions

Target concentrations of 3-NOP were fed in five inclusion rates: 0, 50, 75, 100, and 125 mg/kg of DM. The recovery of 3-NOP from the diet was consistent across all four 3-NOP diets, averaging 64.0% ± 2.98% of the target inclusion rate.

**Figure 2.**
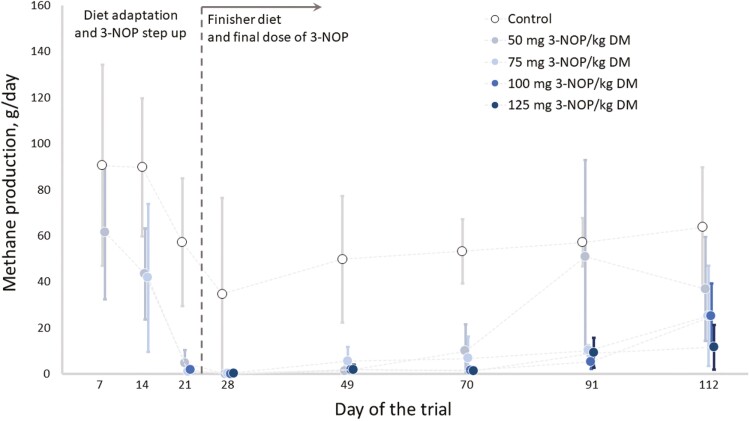
Descriptive statistics (average ± SD) of the CH_4_ production of Angus steers during the adaptation and finisher periods.

CH_4_ production (g CH_4_/d) decreased rapidly during the adaptation period, from days 7 to 28 (Figure 2): CH_4_ production declined 53.4% in the control steers (0 mg 3-NOP/kg DM), and around 99.0% in those steers fed any 3-NOP inclusion rate (Figure 2).

In the finisher period, CH_4_ production (g/d) declined at decreasing rates (i.e., quadratic effect; Table 2), as formulated 3-NOP inclusion rate increased from 0 up 125 mg/kg DM (*P* < 0.01). Tested 3-NOP inclusion rates (50, 75, 100, and 125 mg/kg of DM) resulted in 64.8%, 79.7%, 84.9%, and 90.0% reduction in CH_4_ production respectively, relative to the control diet (0 mg of 3-NOP/kg DM; Table 2). Additionally, CH_4_ production increased as days on feed progressed (*P* < 0.01; Table 2; Figure 2) in the finisher period, with the mean across all treatment groups increasing from 7.73 to 12.4, 16.6, 27.9, and 50.4 g of CH_4_/d from period 1 to 5 of the finisher period, respectively. No interaction between 3-NOP inclusion rate × periods was observed for CH_4_ production (*P* = 0.35), indicating a consistent pattern of mitigation across all 3-NOP inclusion rates over time. Moreover, 3-NOP consistently reduced CH_4_ across hours within a day (please refer to the Supplementary Data).

**Table 2.** Enteric CH_4_ emissions, postfeeding rumen fermentation parameters, and intake (DMI) in Angus steers fed fixed doses of 3-NOP during the five open-circuit respiration chambers runs of the finisher period (days 22 to 112)

Trait^1^	3-NOP dose (mg/kg DM)					SEM^2^	*P*-value^3^			
	0	50	75	100	125		3-NOP		Period	P × 3-NOP
							Linear	Quadratic		
DMI, kg^4^	10.2	10.0	10.2	10.1	9.66	0.159	0.08	0.18	<0.01	0.18
CH_4_ production, g/d	63.7	22.4	12.9	9.63	6.38	4.95	<0.01	<0.01	<0.01	0.76
CH_4_ yield, g/kg DMI	6.21	2.14	1.23	0.912	0.774	0.503	<0.01	<0.01	<0.01	0.82
Rumen pH	6.57	6.59	6.59	6.93	6.55	0.267	0.61	0.72	0.03	0.93
Redox potential (mV)	−168	−180	−186	−164	−170	21.5	0.91	0.43	<0.01	0.87
Rumen ammonia, mg NH_3_/L	41.9	40.0	43.5	34.4	50.24	8.43	0.70	0.45	<0.01	0.73
Total VFA, mmol/L	72.8	83.6	84.3	67.9	79.8	9.29	0.89	0.39	<0.01	0.64
Acetate, mol/100 mol	55.2	57.0	56.5	57.6	58.8	1.72	0.13	0.87	<0.01	0.33
Propionate, mol/100 mol	26.8	25.8	24.7	23.4	24.2	2.14	0.12	0.81	<0.01	0.07
Butyrate, mol/100 mol	9.64	8.97	9.47	9.90	8.75	0.793	0.70	0.86	<0.01	0.51
Acetate:propionate	2.30	2.91	2.75	3.00	3.08	0.428	0.16	0.72	<0.01	0.13

^1^Finisher period from days 22 to 112: five 24 h open-circuit respiration chamber runs on days 28, 49, 70, 91, and 112; DMI recorded on the day of each chamber run; rumen parameters recorded on the day after each chamber run.

^2^SEM = standard error of the mean.

^3^The main effect of 3-NOP dose was decomposed into linear and quadratic orthogonal contrasts and pairwise comparison was performed using Fisher’s protected LSD.

^4^DMI measured in respiration chamber.

CH_4_ yield (g/kg DMI) showed similar behavior to that observed for CH_4_ production being affected by 3-NOP inclusion rate and period (*P* < 0.01), but not by their interaction (*P* = 0.31; Table 2). CH_4_ yield also displayed a quadratic decrease as 3-NOP inclusion rate increased from 0 to 125 mg/kg DM (*P* < 0.01; Table 2). Compared to the control diet, 50, 75, 100, and 125 mg of 3-NOP/kg DM resulted in 65.5%, 80.2%, 85.3%, and 87.6% reduction in CH_4_ yield, respectively (*P* < 0.01; Table 2). From periods 1 to 5 mean CH_4_ yield reduced from 0.711 to 1.36, 1.49, 2.39, and 3.15 g/kg DMI across all treatment groups, respectively.

**Figure 3.**
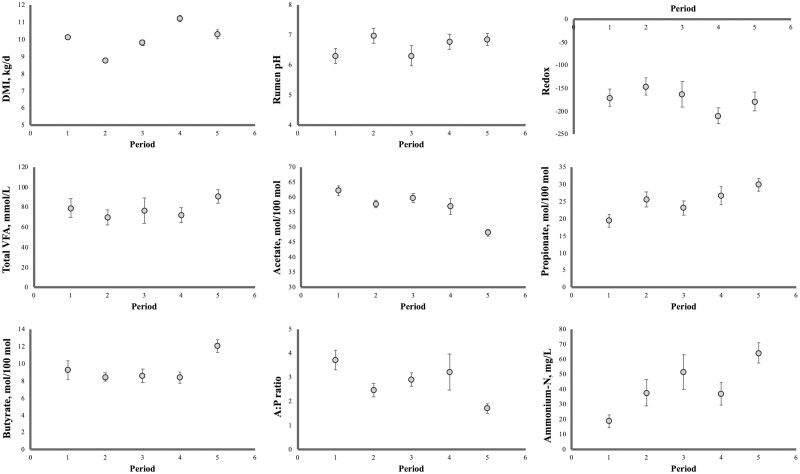
DMI and postfeeding rumen fermentation parameters in Angus steers fed fixed doses of 3-NOP during the five open-circuit respiration chambers runs of the finisher period (days 22 to 112).

Similar to the Dry matter intake (DMI) observed during the 90-d finisher period, during the five open-circuit respiration chambers runs of the finisher period (days 22 to 112), DMI was not influenced by 3-NOP inclusion rate nor 3-NOP × period (*P* ≥ 0.08) interaction (Table 2). However, DMI in chambers varied over time, with means of 10.1, 8.78, 9.80, 11.2, and 10.3 kg recorded in sampling periods 1 to 5, respectively (*P* < 0.01; Figure 3).

### Animal performance

All steers started the transition period at similar BW (*P* = 0.82; Table 3). Supplementation during a 90-d finished period with five inclusion rates of 3-NOP did not affect BW at the end of the transition period (day 21 BW), final BW, DMI, average daily gain (ADG) nor gain to feed ratio (G:F; *P* ≥ 0.36; Table 3).

**Table 3.** DMI, BW, ADG, G:F in Angus steers fed different 3-NOP doses during the finisher period (days 22 to 112)

Trait	3-NOP dose (mg/kg DM)					SEM^5^	*P*-value^6^
	0	50	75	100	125		
Initial BW, kg^1^	354	356	356	355	359	8.04	0.82
Day 21 BW, kg	385	398	394	392	392	8.14	0.64
Final BW, kg	558	561	560	563	567	9.60	0.96
DMI, kg^2^	10.5	10.4	10.5	10.5	10.3	0.139	0.87
DMI, % BW^2^	2.22	2.17	2.19	2.19	2.15	0.0268	0.33
ADG, g/d^3^	1.92	1.81	1.85	1.90	1.95	0.0808	0.69
G:F^4^	0.183	0.173	0.173	0.182	0.189	0.00680	0.36

^1^BW at the start of the adaptation period (day 0). Steers were fed starter diet from days 0 to 7, Intermediate I from days 8 to 14, Intermediate II from days 15 to 21, and Finisher from days 22 to 112.

^2^DMI from days 22 to 112 relative to mean BW for the same period.

^3^ADG from days 22 to 112 (finisher period).

^4^G:F from days 22 to 112 (finisher period).

^5^SEM = standard error of the mean.

^6^When *P*-value for 3-NOP dose was significant (≤ 0.05) pairwise comparison was performed using Fisher’s protected LSD.

### Rumen physiological parameters

Overall, rumen fermentation parameters were not altered by 3-NOP inclusion rates (*P* ≥ 0.12; Table 2). Total VFA production pattern suggests an increase across sampling periods, with measured butyrate and propionate molar proportion being the highest and acetate molar proportion being the lowest at sampling period 5 (*P* < 0.01; Figure 3). Less abundant VFA such as isobutyrate, isovalerate, valerate, and caproate molar concentration did not vary with 3-NOP inclusion rate (*P* ≥ 0.34) nor 3-NOP × period (*P* ≥ 0.35) interaction (VFA means of 0.443 ± 0.0556, 0.491 ± 0.0969, 4.11 ± 0.300 and 3.66 ± 0.402, respectively).

Ammonium-N concentration also increased as days on feed progressed, at the same time that A:P decreased (Figure 3i). No effect on redox potential was observed.

## Discussion

The present study sought to describe and explain the effect of 3-NOP inclusion rate in feedlot cattle related to CH_4_ production and yield, as well as on rumen parameters and steer performance. The results presented here confirmed the consistent effectiveness of 3-NOP in reducing daily CH_4_ emissions in ruminants as shown by the existing literature (Dijkstra et al., 2018; Kim et al., 2020; Almeida et al., 2021). The achieved CH_4_ yield mitigation in our study ranged from approximately 65.5% up to 87.6% relative to control steers fed a finishing feedlot diet. To our knowledge, this is the first experiment under feedlot dietary conditions to report this magnitude of percentage CH_4_ mitigation with 3-NOP, although it should be noted that cattle were fed slightly below ad libitum intake at 2.7 MER during the feeding period.

It is well-recognized that diet composition and inclusion rate of 3-NOP may influence the CH_4_ mitigation achieved, with an increase in CH_4_ reduction observed with increasing inclusion rate of 3-NOP and negative interaction observed with NDF level in the diet (Dijkstra et al., 2018; Kim et al., 2020; Almeida et al., 2021). Diet composition may explain the high percentage mitigation potential of 3-NOP achieved in this study, especially when considering the type of grain, combination of high starch/low NDF, relatively high fat, and inclusion of monensin in the diet. The greatest CH_4_ reduction from 3-NOP previously reported was 81% in a dry-rolled barley based finishing diet, feeding a higher inclusion rate of 3-NOP than that used in our study (200 mg/kg DM, Vyas et al., 2016), however, it should be noted that the diet used in Vyas et al., (2016) did not contain monensin nor any canola meal or canola oil. Vyas et al (2018b) investigated the effect of combining 3-NOP and monensin in both backgrounding and finishing diet but did not find any significant interaction between 3-NOP and monensin, although numerically the CH_4_ yield was further decreased with the combination. Recently, Alemu et al. (2021a) fed a steam-flaked corn-based finisher diet containing 125 mg/kg DM 3-NOP plus monensin, and observed a 76% decrease in CH_4_ yield further supporting that there are several variables within the diet, as well as inclusion rate of 3-NOP, that may influence the percentage CH_4_ mitigation achievable.

Using a meta-analytical approach over 3-NOP inclusion rates between 0 and 337.8 mg/kg DMI in sheep, beef, and dairy cattle, it was previously suggested that 3-NOP inclusion rate resulted in a linear reduction in CH_4_ yield of 0.37 g/kg DMI per milligram of 3-NOP per kg of DM (Kim et al., 2020), however, the pattern of CH_4_ reduction observed in the current study was quadratic, with decreasing rates of CH_4_ reduction as 3-NOP increased to 125 g 3-NOP/kg DMI (Table 3). This would imply that under certain dietary conditions, inclusion rates lower than 125 g 3-NOP/kg DM may be more optimal.

While the proportional reduction of CH_4_ production far exceeds the average mitigation of previous research testing 3-NOP across a range of ruminant diets, this needs to be considered using a GHG inventory perspective in the context of the basal diet and the baseline (i.e., control; Dijkstra et al., 2018) CH_4_ production. Considering the values found in the present feedlot study (i.e., up to 90% reduction from a baseline CH_4_ production of 63.7 g/d), the achievable CH_4_ reduction would be up to 57.3 g/d for feedlot cattle from a baseline CH_4_ production of 63.7 g/d. In contrast, previous literature that tested 3-NOP CH_4_ abatement potential in production systems that use higher roughage in their diets, such as dairy cattle and backgrounding cattle, the achieved reduction in grams per day is higher than that observed in feedlots. For instance, diets averaging 59% roughage in dairy cattle reported around 30% CH_4_ mitigation relative to the baseline CH_4_ (360 g CH_4_/day; averaging values reported by Haisan et al. 2014; Hristov et al., 2015; Lopes et al., 2016; Van Wesemael et al., 2019; Melgar et al. 2020a, 2020b, 2021; van Gastelen et al. 2020, 2022; Yanibada et al., 2020), representing an average daily CH_4_ reduction of 108 g/d. The average reported 3-NOP CH_4_ abatement observed in backgrounding diets with average roughage content of 70%, was 22% reduction from baseline CH_4_ of 176 g CH_4_/d (averaging values reported by Romero-Perez et al. 2014; Vyas et al. 2016, 2018a, 2018b; Kim et al., 2019), representing an average daily CH_4_ reduction of 39 g/d, similar to the absolute abatement achieved in the ­present study. CH_4_ abatement achievable by 3-NOP may, thus, depend on the basal diet.

As an analogue of the methyl-coenzyme M, which is a substrate of the coenzyme M reductase (MCR), involved in the last step of methanogenesis (Duin et al., 2016), 3-NOP has a rapid (Figure 2) and targeted role in inactivating the MCR. This inactivation can be reversed by methanogens which have an endogenous repair system that can re-activate MCR under certain conditions (Prakash et al., 2014). Due to this capability of methanogens, the possibility of their adaptation to the effect of 3-NOP over time is worthy of consideration. Previously, Vyas et al (2016) carried out a long-term feeding study (238 d), taking animals through both backgrounding and finishing diets, and found no adaptation to the use of 3-NOP. In the present study, the 4.5-fold increase in CH_4_ production and yield with progressive days on feed (one to five over the finishing phase) was maintained across all five 3-NOP levels, including Control (0 mg/kg DM 3-NOP), suggesting an increase in CH_4_ emissions that was driven by growth and intake and an adaptation of the rumen microbiota to the basal diet rather than to 3-NOP. This was demonstrated by a nonsignificant 3-NOP by period interaction (Figure 2; Table 3). The increase in CH_4_ in the later feeding periods was followed by a moderate shift in molar proportion of VFAs from acetate towards propionate and butyrate, accompanied by an increase in total VFA production and ammonium-N towards the end of the feeding period.

One important question that remains unanswered after examining the VFA production and molar proportion in the present study is the fate of the spare hydrogen when feeding 3-NOP to feedlot cattle. Previous studies have reported that the blockage of the MCR activity by 3-NOP would result in a build-up of ruminal H_2_ that would cause a shift in VFA molar proportion resulting in less acetate and favoring greater propionate (major hydrogen sink) and butyrate production (alternative and minor hydrogen sink) in the rumen (Romero-Perez et al., 2014; Vyas et al. 2016, 2018a; Alemu et al., 2021b). However, our results revealed that 3-NOP inclusion rate did not alter either total VFA nor VFA molar proportions, thus different rumen H_2_ dynamics may have taken place either involving increased enteric H_2_ emission by the steers and/or rerouting hydrogen into alternative hydrogen sinks such as polyunsaturated fatty acids biohydrogenation pathways (Alemu et al., 2021a), reductive acetogenesis, sulfate, nitrate, and nitrite reduction. The high-concentrate, tempered barley-based diets in the current experiment contained canola oil, with total dietary fat formulated at 7.0% DM. Canola oil is naturally rich in oleic acid C18:1, linoleic acid C18:2, and linolenic acid C18:3 possibly providing a sink for excess hydrogen (see Supplementary Material Table 2). Recently, Zhang et al. (2021) reported that in cattle fed with a barley silage-based diet, the increase in enteric H_2_ yield due to feeding 200 mg/kg DM 3-NOP was lower when it was combined with 50 g/kg DM canola oil. In the study of Alemu et al., 2021a, where a 76% reduction in CH_4_ yield was achieved with 125 mg/kg DM 3-NOP, cattle were fed a steam-flaked corn-based diet plus monensin that had a 4.3% DM endogenous concentration of fat. Corn oil is similarly high in polyunsaturated fatty acids (Barrella-Arellano et al., 2019). Perhaps future microbiome studies could shed some light on the H_2_ dynamics of animals fed this novel feed additive in high concentrate diets and its interaction with dietary fat source and level. From the ciliate protozoa enumeration, we could not draw any conclusion as after the adaptation phase (day 22), no ciliate protozoa were found in the rumen liquid (data not shown). A reduction or absence of ciliate protozoa could be expected when feeding a high-grain diet, as used in this study.

In previous studies in feedlot cattle fed 3-NOP inclusion rates (from 0 up to 200 mg of 3-NOP/kg DM), mixed results have been reported on the effect of 3-NOP on DMI during the finisher period. Small and large pen feedlot experiments conducted by Vyas et al. (2016), 2018b) and Alemu et al. (2021b) reported a reduction in DMI followed by increased G:F, whilst other studies with Latin square designs reported no effect of 3-NOP on DMI of finishing feedlot cattle (Vyas et al., 2018b; Kim et al., 2019). The main purpose for utilizing 3-NOP is to reduce CH_4_ emissions rather than as a performance enhancer; however, assuming that CH_4_ mitigation would result in spare energy (considering same gross energy intake and digestibility; Vyas et al., 2016), it is possible that excess H_2_ could be diverted into sinks that could improve performance in ruminants (Trei et al., 1972; Abecia et al., 2012; Hristov et al., 2015). The present study was designed to investigate the potential CH_4_ mitigating effect of 3-NOP on a high-grain diet typically fed in Australian commercial feedlot systems, whilst monitoring DMI, ADG, and G:F. No adverse effects on DMI, ADG nor F:G were observed. To enable further conclusions on the impact of high CH_4_ reductions on animal performance, larger scale studies should be conducted using a similar diet.

It is noteworthy to mention that in the present study all tested diets contained 25 mg/kg DM of monensin, low roughage levels and added fat. However, recent research (Gruninger et al., 2021; Zhang et al., 2021) suggested that the combination of 3-NOP with other mitigation strategies has the potential to have an additive effect. Nevertheless, future studies should explore possible additive or synergistic effects of combining dietary mitigation strategies with different modes of action to achieve the optimum mitigation potential as well as investigating any potential interactions with other commonly used feed additives and dietary ingredients.

This research has demonstrated that the adoption of 3-NOP may provide a means to mitigate ruminant enteric CH_4_ production and lower the carbon footprint of feedlot cattle production.

## Conclusion

Examining the effect of 3-NOP inclusion rate on CH_4_ emission, rumen parameters, and feedlot steer performance over 112 d on feed (21 d of adaptation plus 90 d on finisher diet), we observed that increasing inclusion rates of 3-NOP up to 125 mg/kg DM reduced both production and yield of CH_4_ in a quadratic manner, by up to 90% during a 90-d finishing period on a tempered barley diet containing monensin and 7% lipid content. Although this experiment was not designed to focus on feedlot performance traits, no detrimental effect of 3-NOP administration was detected on the production parameters measured (i.e., DMI, BW, ADG, G:F).

## Supplementary Material

skad237_suppl_Supplementary_Figure_S1

skad237_suppl_Supplementary_Data

## Abbreviations:

A:P: acetate to propionate ratio
ADF: acid detergent fiber
ADG: average daily gain
BW: body weight
CART: Centre for Animal Research and Teaching
DM: dry matter
DMI: dry matter intake
G:F: gain to feed ratio
GHGs: greenhouse gases
MER: maintenance energy requirement
ME: metabolizable energy
CH_4_: methane
NDF: neutral detergent fiber
VFA: volatile fatty acids
3-NOP: 3-nitrooxypropanol
