# Supplementary figures and images for: Effect of 3-nitrooxypropanol on enteric methane emissions of feedlot cattle fed with a tempered barley-based diet with canola oil
Source: J Anim Sci. 2023 Jul 10;101:skad237. doi: 10.1093/jas/skad237 (PMC10370881; doi:10.1093/jas/skad237)

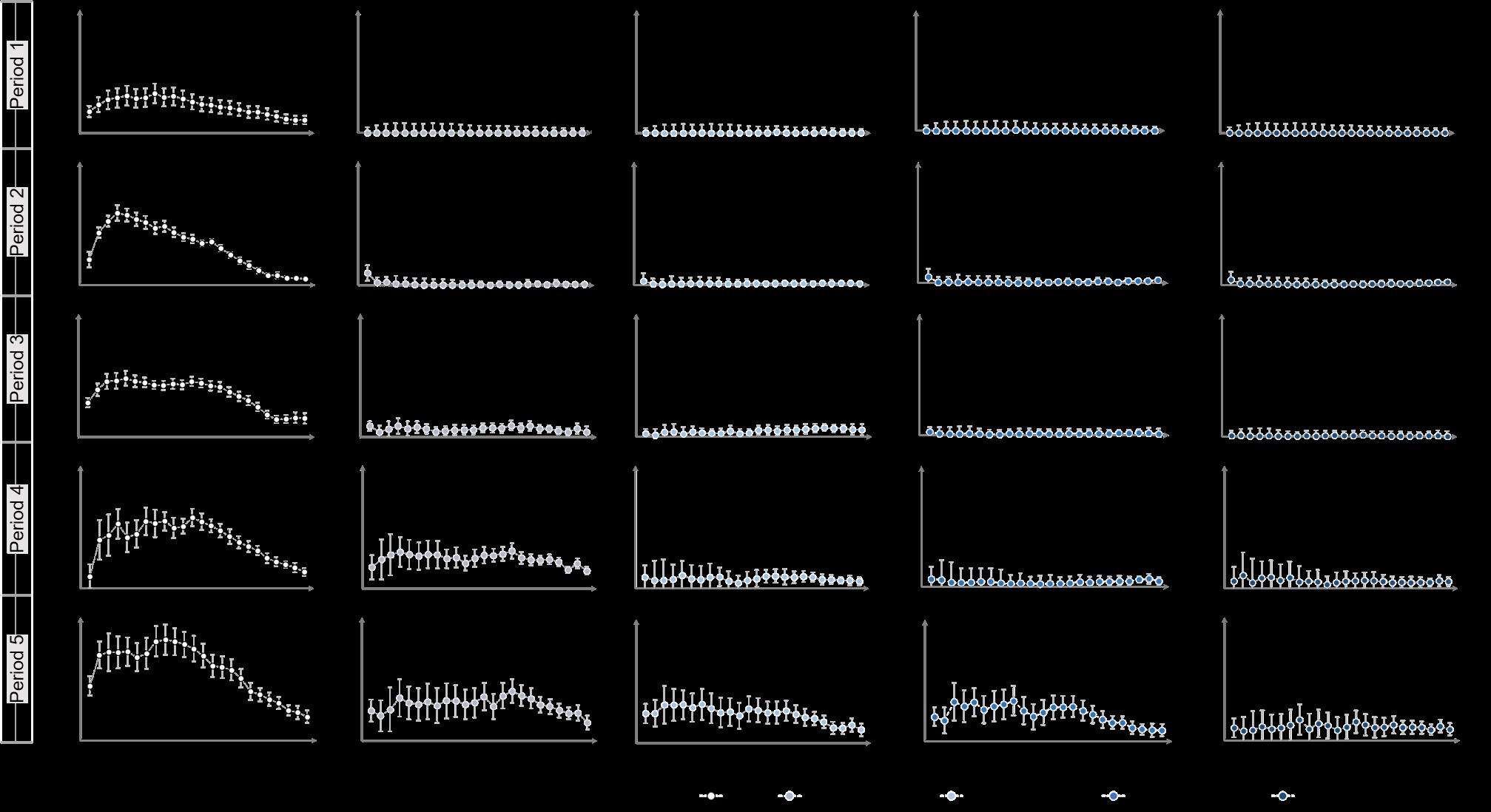

Supplement: skad237_suppl_Supplementary_Figure_S1 [file skad237_suppl_supplementary_figure_s1.jpeg]
