# Supplementary material for: Effect of 3-nitrooxypropanol on enteric methane emissions of feedlot cattle fed with a tempered barley-based diet with canola oil
Source: J Anim Sci. 2023 Jul 10;101:skad237. doi: 10.1093/jas/skad237 (PMC10370881; doi:10.1093/jas/skad237)

Running Head: 3-NOP on enteric methane emissions

**Effect of 3-nitrooxypropanol on enteric methane emissions of feedlot cattle fed a tempered barley-based diet with canola oil ^1^**

A. K. Almeida^*§2^, F. Cowley^*^, J.P. McMeniman^†^, A. Karagiannis^‡^, N. Walker^‡^, L. F. M. Tamassia^‡^, J. J. McGrath^‡*^, R. S. Hegarty^*^

^*^School of Environmental and Rural Science, University of New England, Armidale, NSW 2351, Australia

^†^ Meat & Livestock Australia Limited (MLA), 40 Mount Street, North Sydney, NSW 2060, Australia

^‡^ DSM Nutritional Products, Wurmisweg 576 4303 Kaiseraugst Switzerland

§ School of Agriculture & Environment, Massey University, Palmerston North 4442, New Zealand.

^1^We thank Meat Livestock Australia (MLA, project code: B.FLT.5010) for providing financial support to conduct this research.

^2^Corresponding author: almeida.amelia@gmail.com

# SUPPLEMENTARY MATERIAL

Table 1. Pre-feeding rumen fermentation parameters in Angus steers fed increasing doses of 3-NOP during the five open-circuit respiration chambers runs of the finisher period (day 22 to 112).

| Trait ^1^ |  | 3-NOP dose (mg/kg DM) | | | | |  | SEM^2^ | P-value^3^ |  |  |  |
| --- | --- | --- | --- | --- | --- | --- | --- | --- | --- | --- | --- | --- |
|  |  | 0 | 50 | 75 | 100 | 125 |  |  | 3-NOP |  | Period | P×3-NOP |
|  |  |  |  |  |  |  |  |  | Linear | Quadratic |  |  |
| Rumen pH |  | 8.03 | 8.09 | 8.11 | 7.93 | 7.98 |  | 0.101 | 0.45 | 0.37 | <0.01 | 0.20 |
| Redox potential |  | -171 | -191 | -164 | -185 | -201 |  | 18.1 | 0.33 | 0.58 | <0.01 | 0.66 |
| Ammonium-N (mg N/L) |  | 92.9 | 114 | 91.0 | 95.1 | 98.1 |  | 11.8 | 0.93 | 0.48 | <0.01 | 0.86 |
| Total VFA (mmol/L) |  | 34.7 | 29.1 | 30.1 | 32.4 | 34.9 |  | 2.89 | 0.91 | 0.74 | <0.01 | 0.32 |
| Acetate (mol/100 mol) |  | 56.8 | 56.0 | 57.0 | 55.7 | 56.6 |  | 1.28 | 0.86 | 0.82 | 0.01 | 0.77 |
| Propionate (mol/100 mol) |  | 24.0 | 24.6 | 25.1 | 23.2 | 24.5 |  | 1.50 | 0.98 | 0.79 | <0.01 | 0.15 |
| Butyrate (mol/100 mol) |  | 10.3 | 9.56 | 8.88 | 11.0 | 9.35 |  | 0.772 | 0.74 | 0.60 | 0.01 | 0.95 |
| Acetate:propionate |  | 2.56 | 2.64 | 2.42 | 2.67 | 2.55 |  | 0.223 | 0.97 | 0.99 | <0.01 | 0.42 |

^1^Finisher period from d 22 to 112: five 24-h open-circuit respiration chamber runs on d 28, 49, 70, 91 and 112; DMI recorded on the day of each chamber run; rumen parameters recorded on the day before each chamber run; ^2^SEM = standard error of the mean. ^3^The main effect of 3-NOP dose was decomposed into linear and quadratic orthogonal contrasts and pairwise comparison was performed using Fisher's protected LSD, in that case means within a row showing different superscripts depict significant effects of 3-NOP dose, period and interaction.

Table 2. Fatty acid profile of the vegetable oil added to the feedlot total mixed rations feed to Angus steers during the 112-d feeding period.

| Fatty acid | % |
| --- | --- |
| Myristic acid C14:0 | 0.07 |
| Palmitic acid C16:0 | 4.4 |
| Palmitoleic acid C16:1 | 0.3 |
| Heptadecanoic acid C17:0 | 0.1 |
| Heptadecenoic acid C17:1 | 0.1 |
| Stearic acid C18:0 | 2.1 |
| Oleic acid C18:1 | 62.3 |
| Linoleic acid C18:2 | 18.4 |
| Linolenic acid C18:3 | 10.1 |
| Arachidic acid C20:0 | 0.6 |
| Eicosenoic acid C20:1 | 1.0 |
| Behenic acid C22:0 | 0.3 |
| Erucic acid C22:1 | <0.1 |
| Lignoceric acid C24:0 | 0.1 |
| Tetracosenoic acid C24:1 | 0.1 |
| Total Fatty Acids | 100 |

^Analysis performed by NSW DPI Laboratory Services - Wagga Wagga Chemistry Services Laboratory, PMB Pine Gully Road, Wagga Wagga NSW 2650. Edible Oil Fatty Acids Profile: 2-1702.^

Figure 1. Hourly methane production (g) Angus steers fed specified doses of 3-NOP during the five open-circuit respiration chambers runs of the finisher period (d 22 to 112), each line depicts one period.


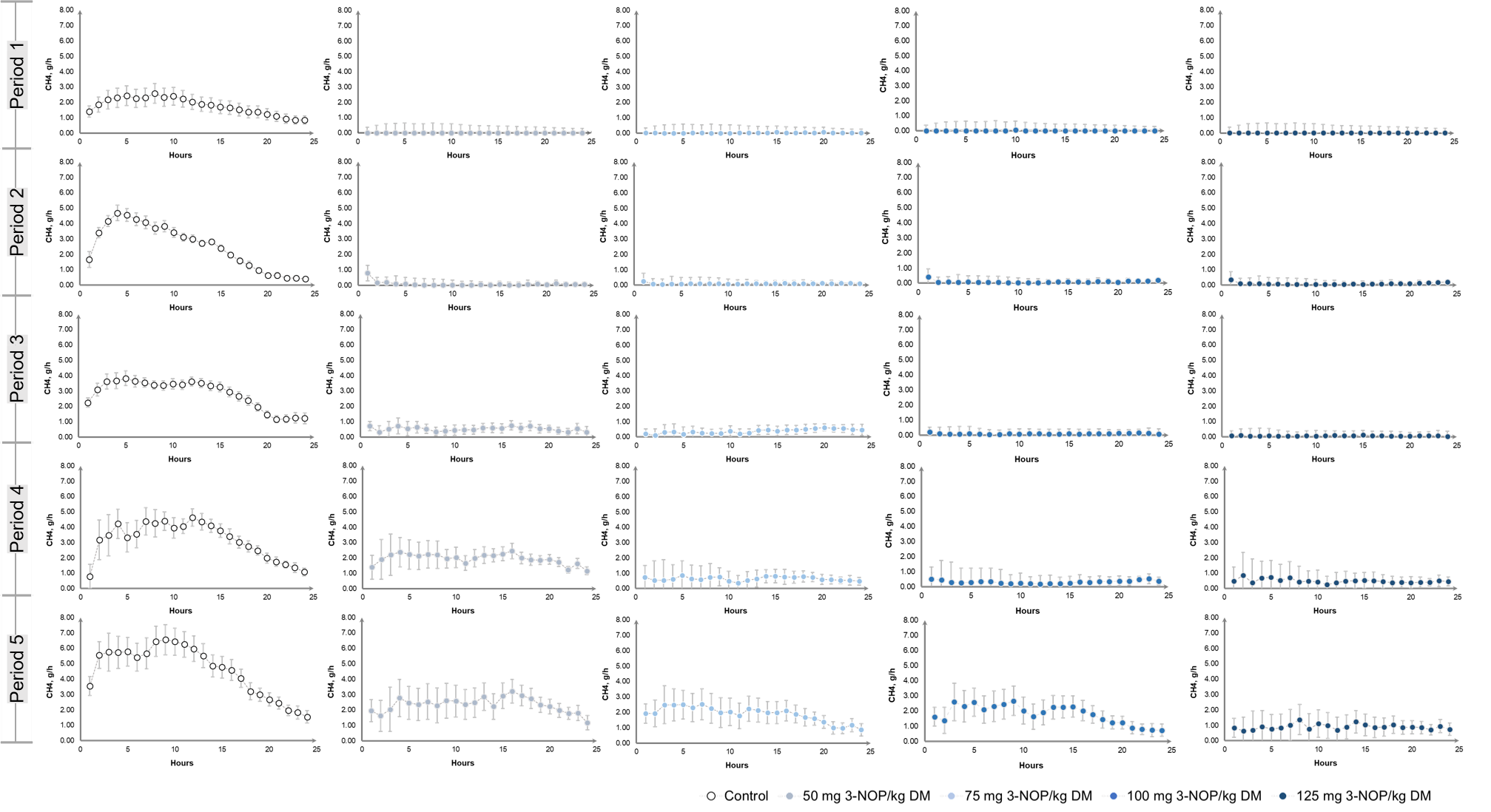

Supplement: skad237_suppl_Supplementary_Data [file skad237_suppl_supplementary_data.docx]
